# Mental health diagnoses in adults with phenylketonuria: a retrospective systematic audit in a large UK single centre

**DOI:** 10.1186/s13023-021-02138-z

**Published:** 2021-12-20

**Authors:** George Altman, Kamran Hussain, Diane Green, Boyd J. G. Strauss, Gisela Wilcox

**Affiliations:** 1 Faculty of Biology, Medicine and Health, University of Manchester, Manchester, UK; 2 The Mark Holland Adult Inherited Metabolic Disorders Unit, Ladywell Building, Salford Royal NHS Foundation Trust, Stott Lane, Salford, M6 8HD UK; 3 Department of Medicine, School of Clinical Sciences, Faculty of Medicine, Nursing and Health Sciences, Monash University, Melbourne, Australia

**Keywords:** Phenylketonuria, Phenylalanine, Neuropsychiatric, Mental health, Depression, Anxiety, Mood swings, Low mood, Audit, ESPKU guidelines

## Abstract

**Background:**

Recently published European Society for Phenylketonuria (ESPKU) guidelines have recommended a lifelong diet with phenylalanine (Phe) control ≤ 600 μmol/L for phenylketonuria (PKU) patients. This study aimed to identify whether PKU adult patients are at a higher risk of mental health diagnoses if their 2-year average Phe level is higher than the ESPKU European guidelines. Published studies identified by a literature review showed that related studies have been published in American and European PKU study populations but not in the United Kingdom (UK) study populations. Previous studies also involved a smaller number of participants due to this being a rare disease.

**Results:**

We undertook a retrospective audit at a single large PKU centre in the UK. 244 adult PKU patients at the centre were included, 220 of which had a recorded Phe level. Approximately 75% of the patients in this study did not meet the ESPKU European guidelines for Phe control. A systematic search of the electronic patient record was undertaken looking for mental health diagnoses. Compared to two-year average Phe levels ≤ 600 μmol/L, PKU adult patients with two-year average Phe levels > 600 μmol/L were more likely to have diagnoses of low mood, depression, anxiety, or mood swings, but only low mood reached statistical significance (*p* < 0.05).

**Conclusions:**

PKU patients with two-year average Phenylalanine levels greater than ESPKU guidelines may be at greater risk of mental health diagnoses and symptoms. Many of these adult PKU patients will be lost to follow-up, and therefore may be receiving treatment for mental health conditions in the community. Multicentre UK studies and international collaborations are required to overcome low participant numbers in the study of this rare disease.

## Background

Phenylketonuria (PKU) is an autosomal recessive inherited metabolic disorder. Patients with the condition have an absent or dysfunctional phenylalanine hydroxylase (PAH) [1]. The deficiency in PAH results in high phenylalanine (Phe) levels in the blood. Excess phenylalanine is toxic to the developing brain and competes with other large neutral amino acids e.g. tryptophan for crossing the blood–brain barrier. Together with deficient tyrosine, this causes marked neurotransmitter derangement, with deficiencies of dopamine, noradrenaline, and serotonin [2]. In the UK, an estimated 1 in 10,000 newborn babies will have PKU [3]. Currently, the only treatment available for all PKU patients in the UK is a lifelong phe restricted diet. This diet was first conceptualised by Professor Isaac Woolf using casein hydrolysate treated with active carbon to produce a dietary supplement low in Phe [4]. Woolf worked with Dr Horst Bickel to treat the first patient with this diet at Birmingham Children's Hospital [5]. PKU patient outcomes were revolutionised by this therapeutic diet. Extreme natural protein restriction, supplemented with micronutrient-fortified phenylalanine-free amino acid-based supplements to meet nutritional requirements is instituted neonatally [2]. Close blood-spot monitoring of Phe levels, maintained throughout development, has enabled the attainment of near-potential IQ [6]. However, adherence to such dietary stringency is difficult for many, and alternative therapies e.g. tetrahydrobiopterin (BH4) or Kuvan®, co-factor for PAH, are limited to those with residual enzyme activity, demonstrated responsiveness, and access [6–9]. Furthermore, it is estimated that half of all patients in the UK with inherited metabolic disease have been lost to follow-up; this means there may be a significant number of patients no longer on the diet, who are receiving care only within a general practice setting [10]. It is essential to determine the impact of high Phe levels on mental health diagnoses, as there may be a considerable unidentified burden of mental health disease in this population.

In 2017, the ESPKU guidelines were published, which advised targeting Phe ≤ 600 μmol/L lifelong, with a lower target during pregnancy and preconception [2]. The evidence level was graded at D; therefore further studies are required to improve the strength of evidence for this guideline. In view of controversy amongst key opinion leaders in the relationship between concurrent Phe control and mental health, and the relative lack of evidence in adults. We sought to investigate, in our large unselected patient cohort, if a relationship could be observed between prevailing phenylalanine control and the prevalence of mental health symptoms [2]. We used a method of keyword searches to extract recorded mental health symptoms as recorded by clinicians in the electronic patient record, a form of real world evidence.

This study asked the following research question: are adult patients with PKU at higher risk of mental health diagnoses if their average Phe level is higher than the ESPKU European Guidelines of ≤ 600 μmol/L? The null hypothesis is that there is no significant difference in the prevalence of mental health diagnoses between patients with a 2-year average Phe level above and below the ESPKU Guidelines of ≤ 600 μmol/L. A single-centre retrospective clinical audit at the Mark Holland Metabolic Unit, Salford Royal NHS Foundation Trust, Manchester, United Kingdom was undertaken.

## Literature review

A literature review was undertaken to identify previous methodologies used to answer this research question. Studies were filtered to include adult patients with a diagnosis of PKU. Relevant outcomes sought were the prevalence of mental health diagnoses in this population (low mood, anxiety, depression, and mood swings). Published studies in the last 30 years, investigating the relationship between Phe levels and mental health diagnoses were included in the literature review.

The search strategy involved a database search using a scoping review. Boolean logic was used to combine keywords: Depression, Anxiety, Mood, PKU, Phenylketonuria, Phenylalanine. The search utilised three databases: Pubmed, Google Scholar, and the University of Manchester library. The final method for identifying additional papers was citation chaining.

**Fig. 1:**
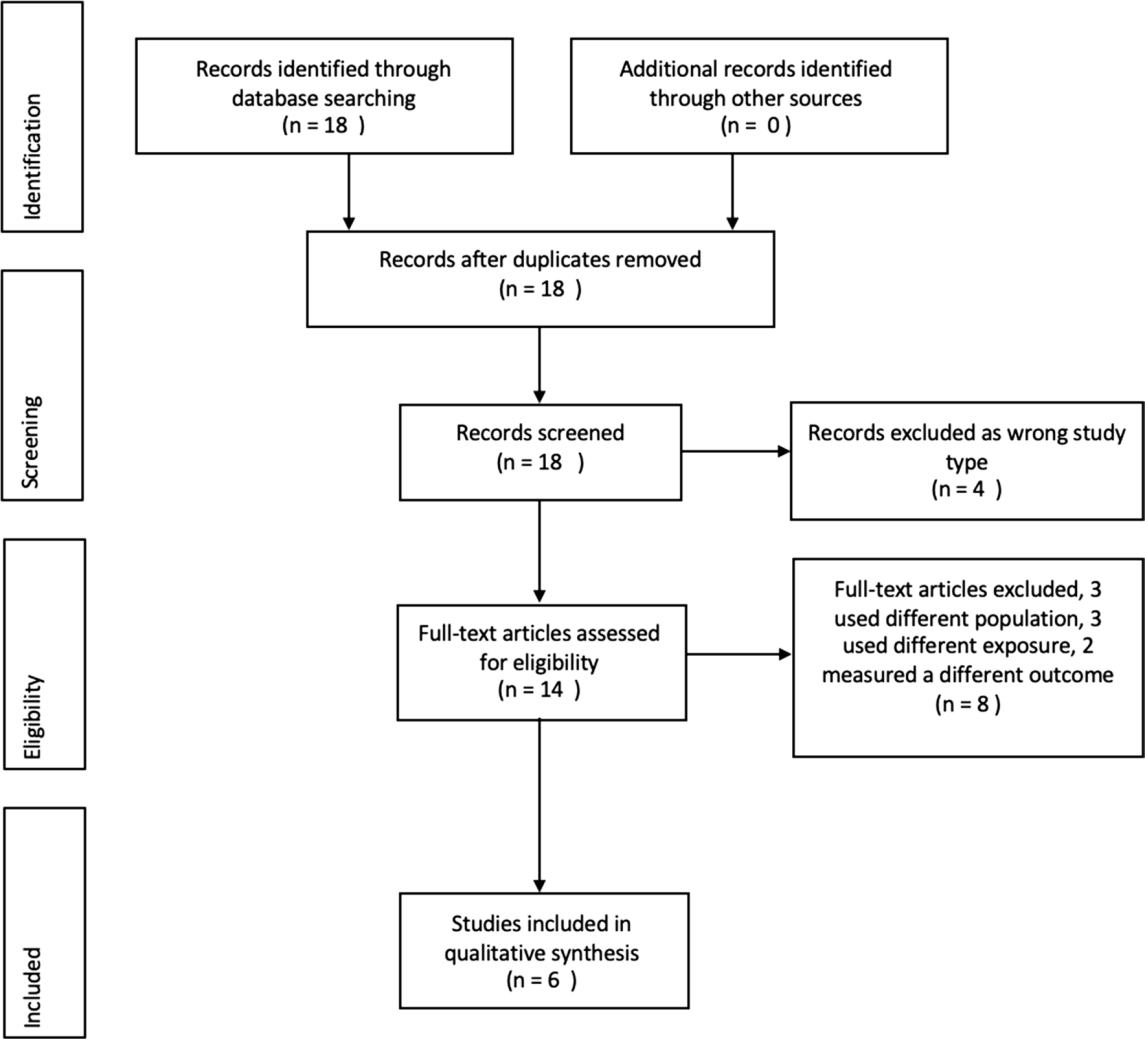
Adapted from PRISMA Flow chart for literature search—from: Moher D, Liberati A, Tetzlaff J, Altman DG, The PRISMA Group (2009). Preferred reporting items for systematic reviews and meta-analyses: The PRISMA statement. PLoS Med 6(7): e1000097. 10.1371/journal.pmed1000097

Six papers met the search criteria [11–16]. In terms of validity, there was a wide range of study designs. In terms of reliability, two studies found a significant relationship between Phe levels and neuropsychiatric symptoms [11, 12]; one study found a non-statistically significant relationship and three studies found no relationship [13–16]. In terms of applicability, none of the studies included patients from the UK, which limits the application of these results to a UK PKU population (Fig. 1).

## Method of audit

A systematic registered audit was undertaken using the clinical database maintained by the Mark Holland Metabolic Unit at the Salford Royal NHS Foundation Trust. The database contained 244 adult patients with PKU; 24 patients had no recorded Phe levels in the last two years and were excluded from the audit. Seventeen patients had a pregnancy during the previous two years; an additional separate analysis of pregnant PKU patients was included since pregnant patients have different targets regarding Phe control(< 360 μmol/L).

The 2-year average Phe level was used as the measure of Phe control. Calculation of the average used all measurements recorded on the electronic patient record, or clinic letters, during the previous two years. All outpatient clinical letters in the electronic medical record were combined as a single document and searched in the preceding 2 years for the terms:

“Depression", "Anxiety", "Low Mood" and "Mood swings". This included both longstanding diagnoses recorded in the problem list as well as emerging diagnoses or symptoms recorded in the consultation by the clinician. If a clinician used the terms in a list of diagnoses or a problems list at any point in the 2 years prior to our data collection, this was recorded as a positive result. Patient age, sex, and pregnancy status were also recorded. Odds ratios (with 95% confidence intervals), were calculated to establish the association between Phe level > 600 μmol/L and mental health diagnoses. The data was independently double-checked for accuracy by both first authors.

## Results

Our initial exploratory analysis demonstrated several notable trends throughout the extent of the dataset. The mean age was 35.9 years with a range of 18–71 years. 27 patients were born before 1969 when national heelprick test screening started in the UK. Before this Phenistix nappy testing was used which missed an estimated 25–50% of cases. Therefore it is likely that some of these patients were delayed in starting treatment [17]. The data evidencing the precise extent of such a delay was not available, however, the mean prevalence of mental health symptoms in patients born before heelprick testing was 33.3%, compared with 16.3% in those born after the advent of heelprick testing. The mean number of Phe readings was 7.1 over two years, with a range of 1–70. The mean Phe level was 944.6 μmol/L (standard deviation 443.46 μmol/L) and a range of 162-2003 μmol/L. Forty-nine of 202 patients had a 2-year average Phe ≤ 600 μmol/L i.e. 24.6% of non-pregnant patients were adhering to ESPKU European Guidelines [2]. 9/17 pregnant patients had a two-year average Phe level < 360 μmol/L during pregnancy and preconception. Therefore 52.9% of pregnant patients were adhering to ESPKU guidelines.

### Mental health symptoms

Odds ratios (with 95% confidence intervals), were calculated to establish the association between Phe level > 600 μmol/L and mental health diagnoses. Overall, 31(14.1%) of non-pregnant patients had an electronic medical record diagnosis of depression. 42(19.1%) patients had a diagnosis of anxiety. 32(14.5%) patients had a diagnosis of low mood, and 22(10%) patients had a diagnosis of mood swings.

### Odds ratios including pregnant women

#### Depression

See Table 1.

**Table 1:** Patients with a depression diagnosis in each group of phenylalanine control

Depression diagnosed	Phe > 600 μmol/L	Phe ≤ 600 μmol/L	Total
Yes	26	4	30
No	128	45	173
Total	154	49	203

Odds ratio: 2.9885. 95% CI 1.0001–8.9301. z statistic: 1.960

Significance level *P* = 0.0500

#### Anxiety

See Table 2.

**Table 2:** Patients with a anxiety diagnosis in each group of phenylalanine control

Anxiety diagnosed	Phe > 600 μmol/L	Phe ≤ 600 μmol/L	Total
Yes	34	6	40
No	120	43	163
Total	154	49	203

Odds ratio: 2.2358. 95% CI: 0.9352–5.3450. z statistic: 1.809

Significance level *P* = 0.0704

#### Low mood

See Table 3.

**Table 3:** Patients with a low mood diagnosis in each group of phenylalanine control

Low mood diagnosed	Phe > 600 μmol/L	Phe ≤ 600 μmol/L	Total
Yes	27	4	31
No	127	45	172
Total	154	49	203

Odds ratio: 3.1231. 95% CI: 1.0475–9.3114. z statistic: 2.043

Significance level *P* = 0.0410

#### Mood swings

See Table 4.

**Table 4:** Patients with a mood swings diagnosis in each group of phenylalanine control

Mood swings diagnosed	Phe > 600 μmol/L	Phe ≤ 600 μmol/L	Total
Yes	19	2	21
No	135	47	182
Total	154	49	203

Odds ratio: 4.3478. 95% CI: 0.9850–19.1924. z statistic: 1.940

Significance level *P* = 0.0524

#### Diagnosis/symptom

**Fig. 2:**
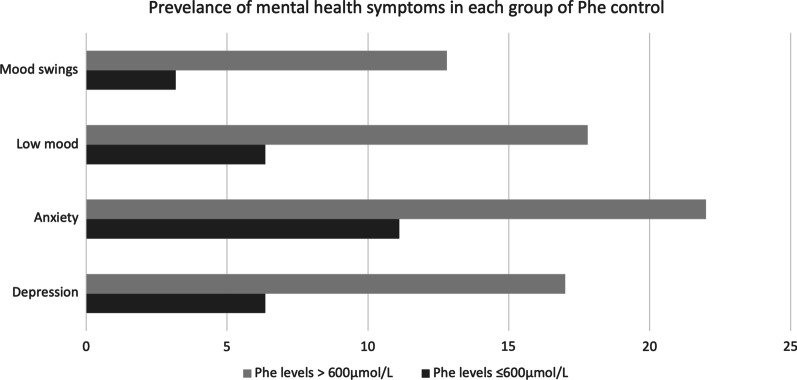
Shows the percentage of patients with mood swings, low mood*, anxiety, or depression in patients with 2-year average Phe levels > 600 μmol/L and ≤ 600 μmol/L. *P < 0.05

See Table 5 and Fig. 2.

**Table 5:** Table showing odds ratios relative to the lowest quartile of Phe levels for each mental health diagnosis, 95% confidence interval, and *P* value

Diagnosis/symptom	Odds ratio	95% CI	Significance
Depression	2.9	1–8.9	*P* = 0.0500
Anxiety	2.2	0.93–5.35	*P* = 0.0704
Low mood	3.1	1.04–9.31	*P* = 0.0410
Mood swings	4.3	0.98–19.1	*P* = 0.0524

## Discussion

### Possible mechanisms of development of mental health conditions in PKU

There are a number of ways in which phenylketonuria may impact neuropsychological functioning and mood. This includes both short-term and long-term effects, both biological and psychosocial.

Long-term developmental effects may result from the cumulative impact of elevated blood phenylalanine competitively disrupting the transport of other large neutral amino acids (LNAA) across the blood brain barrier (BBB) via the LAT1 transporter [2]. These LNAA include essential amino acids required for protein and myelin synthesis as well as tyrosine which can be considered conditionally essential in PKU. Tyrosine and tryptophan, required for dopamine and serotonin synthesis respectively, are reduced in their CNS uptake and furthermore, phenylalanine elevation inhibits the enzymatic activity required to synthesise those specific neurotransmitters. The various biological mechanisms of the neuropsychological impact of PKU are described in further depth and breadth in the comprehensive review by Ashe et al. [18].

Whilst early-treated patients may be significantly protected from the major impact of severe hyperphenylalaninaemia, the effects of elevated phenylalanine levels on neurotransmitter production persist, potentially impacting mood and executive function. These mechanisms may underlie the observations in our unselected PKU patient cohort as well as those reported in the literature [2]. Our findings of a possible relationship between contemporaneous phenylalanine control and clinically recorded mental health symptoms, reaching statistical significance with respect to low mood, might reflect short to medium term effects of hyperphenylalanimaemia but longitudinal studies in such patients might be needed to differentiate short-term versus long-term effects.

Independent of phenylalanine control, significant psychosocial factors are impacting on the mental health of our PKU patients. Some of these relate to the issues of having a rare condition, requiring an unusual and highly restrictive diet, and the intra-familial stress and other social stresses that follow [5]. Our study was not designed to capture these longer-term aspects of PKU.

The current first-line treatment for depression and anxiety is the use of selective serotonin reuptake inhibitors. Due to the reduced availability of serotonin, this first-line treatment may not be as effective in PKU patients, Ashe et al. identified no studies of antidepressant effectiveness in PKU patients in their review article [18].

### Limitations of the current literature

There are several limitations of the currently available literature on the relationship between Phe levels and neuropsychiatric symptoms in PKU. First, there is an over-reliance on studies containing low numbers of participants. This is to be expected with a rare disease. In the future, multicentre studies should be used to overcome this limitation. Second, the literature review does not represent the UK PKU population, since, to our knowledge, no studies on the mental health of PKU patients have been completed in the UK. The age of participants is generally young, which does not represent the entire PKU population. Bilder et al. suggest a positive correlation between age and the presence and intensity of neuropsychiatric symptoms in PKU patients [13]. A young adult study population may risk underestimating the burden of neuropsychiatric symptoms in the whole PKU population. Third, the literature reviewed used a wide range of different questionnaires and screening tools which may not be directly comparable. Last, there is no standard measure of Phe levels with studies using a range of methods (current, 6-month average, 2-year average) which may obscure the relationship between Phe levels and neuropsychiatric symptoms. As the randomised control trial was the strongest form of evidence available for the current literature review, there is strong support for a link between Phe levels and neuropsychiatric symptoms [11]. However, further work needs to be done to consider the impact of a range of confounding factors (socioeconomic status, age, gender, and other biomarkers such as tyrosine and tryptophan levels).

### Results including pregnant patients

All four mental health diagnoses have an odds ratio of > 1, therefore exposure to two-year average Phe of > 600 is associated with higher odds of each outcome (depression, anxiety, low mood, mood swings). The analysis of low mood has a 95% confidence interval of 1.0475–9.3114, demonstrating a significant result. However, there is a low level of precision as the odds ratio range is large. Depression overlaps with 1 but has a significance level of 0.05 thus representing a strong association. With regard to anxiety and mood swings, the association of Phe > 600 and increased prevalence of these conditions did not reach significance but shows a strong trend despite the limitations and exploratory nature of the study.

### Results excluding pregnant patients

All four mental health diagnoses have an odds ratio > 1, therefore exposure to 2 year average Phe of > 600 is associated with higher odds of each outcome (depression, anxiety, low mood, mood swing). Despite this, the association between Phe and mental health symptoms did not reach significance in this group.

### Benefits of the study

The study used audit data of all PKU patients in the clinic database and is thus a form of real-world evidence. Using real-world evidence reduces the likelihood of selection bias [19, 20]. However, patients may still have been selected in some form, as patients will be removed from the database if they miss two clinic appointments, this procedure may have ‘selected’ for more motivated patients who are more likely to engage with healthcare professionals. The evidence is drawn from a wider group of patients than previous studies in the literature review which focused on younger PKU patients aged 20–30. This study has a wider age range which may allow for better appreciation of changes in mental health diagnoses in older PKU patients but may still have missed those PKU patients whose diagnosis may have been missed before the introduction of Guthrie testing in the late 1960s. Ageing in treated PKU patients is currently an area with little evidence available as treated patients are only just reaching their fifth and sixth decade [21]. This study used a larger population of patients than previous studies, giving the study greater power to find relationships between the exposure and outcome. Finally, this study is the first using a UK PKU population, studying this population may identify specific trends in the UK PKU population.

### Limitations of the audit

The comprehensiveness of the dataset with regard to the multitude of factors influencing Phe control prevents us from establishing causality. In addition, there may be an issue of reverse causality due to timelines; this could occur if a PKU patient with depression subsequently commenced a low Phe diet. The patient may still be recorded as having a diagnosis of depression but would now have a lower Phe level. However, Burgess et al. identified improvements in anxiety and depression ratings after the reintroduction of dietary control of PKU showing causality between reduced Phe levels and improving mental health symptoms suggesting reverse causality may be unlikely in this case [22]. However, the direction of the association between Phe level and mental health diagnoses remains uncertain in our patient group.

Several variables were not controlled for and may be confounding including age, gender, socioeconomic circumstances, tyrosine/tryptophan levels. The study also relies on the accuracy of the patient record. There are many sources of error in electronic patient records, including incorrect data entry and the introduction of incorrect diagnoses which are not later removed [23].

There may also be hidden time effects present; if there is seasonal variation in the prevalence of mental health symptoms, then the two-year sampling may not have equally sampled seasons with increased incidence of anxiety or depression [24].

Mental health conditions may be underdiagnosed; patients with borderline or subthreshold anxiety or depression may not be detected in the clinic. Therefore the results may underestimate the prevalence of anxiety and depression.

A final consideration is the impact of diet on mental health. Other chronic diseases which involve restricted diet eg diabetes, have an increased prevalence of mental health diagnoses [25]. The PKU diet is significantly more restricting, and patients may find it time-consuming and socially isolating [5]. Being on a Phe restricted diet may itself be a source of stress and anxiety [26]. This could increase the prevalence of anxiety in patients with low Phe and confound the result.

## Conclusion

In this audit, ≅ 75% of adults Phe control did not meet the ≤ 600 μmol/L target outlines in the European Guidelines. The hypothesis put forward was that patients with PKU were at higher risk of mental health diagnoses if their average Phe levels were higher than the ESPKU guidelines of ≤ 600 μmol/L. There is a statistically significant association between patients exposed to a two-year average Phe > 600 μmol/L and a diagnosis of low mood. PKU patients with Phe > 600 μmol/L were more likely to have a diagnosis of depression, anxiety, or mood swings, but these trends were not statistically significant. However, the underdiagnosis of depression and anxiety in clinical settings may contribute to this outcome. Those whose Phe levels were greater than the guidelines appeared to have greater morbidity with respect to mental health symptoms, but larger studies including a prospective multicentre audit are needed to confirm this observation.

This audit represents the largest in terms of sample size to date, and, to our knowledge, the first to investigate mental health diagnoses in a UK PKU population. The implications for future research are that multicentre studies are required to improve the precision and power of the research. Future studies should aim to include consideration of confounding factors and use study designs that can avoid reverse causality such as a case–control study. In addition, the use of a more detailed dataset including details such as mean time to presentation from diagnosis, socioeconomic status, or region would be of greater utility in establishing causality, beyond the associations we have demonstrated. Expanding the assessment of mental health symptoms to include verified rating scales and diagnostic interviews would enable a greater level of precision when comparing these with the contemporaneously recorded Phe levels.

## Data Availability

Please contact author for data requests.
